# Social support factors associated with psychological resilience among women survivors of intimate partner violence in Gauteng, South Africa

**DOI:** 10.1080/16549716.2018.1491114

**Published:** 2018-10-01

**Authors:** Mercilene T Machisa, Nicola Christofides, Rachel Jewkes

**Affiliations:** a Gender and Health Research Unit, South African Medical Research Council, Pretoria, South Africa; b School of Public Health, University of Witwatersrand, Johannesburg, South Africa

**Keywords:** Resilience, intimate partner violence, mental health; PTSD; depression; trauma, social support

## Abstract

**Background**: Women’s experiences of intimate partner violence (IPV) increase their risk for mental ill health. However, some women exposed to IPV and adversity are psychologically resilient and function well despite these exposures.

**Objectives**: We conducted a study to investigate the factors that are associated with psychological resilience among abused women, using data collected in a household survey conducted in Gauteng province of South Africa.

**Methods**: Data is from a cross-sectional study. A multi-stage random sampling approach was used to select a sample of 501 women. The World Health Organization (WHO) Multi-Country Study on Women’s Health and Domestic Violence Questionnaire was used to measure lifetime experience of physical and sexual IPV. Only 189 women who had experienced lifetime IPV were included in this secondary analysis. Resilience was measured as scoring below the threshold for the Centre for Epidemiological Studies Depression Scale and the Harvard Trauma Questionnaire. Other explanatory factors measured included child sexual abuse, non-partner rape, other traumatic life events, social support indicators, binge drinking and socio-demographic variables. Multivariable regression analysis was used to test factors associated with resilience.

**Results**: Forty two percent of women scored below the threshold for post-traumatic stress disorder (PTSD) or depressive symptoms at the time of the survey and so were categorized as resilient. Social support indicators were associated with increased resilience. Women who perceived that their communities were supportive and they would easily find money in an emergency were more likely to be resilient. Women who binge drank, experienced severe IPV in the past 12 months, received negative reactions to disclosure and utilized medical or psychosocial services were less likely to be resilient.

**Conclusion**: Social support indicators including social connectedness, stronger network ties and perceived supportive communities are key factors in fostering resilience among abused women. Interventions should aim to promote stronger and supportive social networks and increase women’s utilization of formal support services.

## Background

Women survivors of intimate partner violence (IPV) are at increased risk of mental ill health including depression and post-traumatic stress disorder (PTSD) [1–7], notwithstanding the fact that the relationship of IPV and mental ill health may be bidirectional [3–9]. Mental ill health among women has also been partially explained by pathways from other violent exposures, including histories of childhood physical and/or sexual abuse, rape by non-partners or other life traumatic events such as near-death experiences or being victims of crimes [8,10–13]. Multiple and repeat victimization compounds the negative mental health effects experienced by women. Socio-economic factors such as social class and poverty, low levels of education, unemployment and limited social support also negatively impact on women’s mental health [12,14–16].

While women’s IPV experiences increase their risk of mental ill health, some women exposed to IPV, adversity and multiple forms of victimization function well despite these exposures. The absence of psychopathology after traumatic exposure is an indicator of resilience [17]. Pathways to psychological resilience include positive adaptation to adversity including overcoming highly stressful events or trauma or challenging situations and maintaining mental health [18–20]. Psychological resilience is therefore both a dynamic process and an outcome that results from individuals being able to interact with their environments to promote mental well-being or protect themselves against the influence of adverse risk factors [20]. In the context of social support, psychological resilience entails the ability to harness key supportive resources, which may be emotional, informational or practical in nature, in order to sustain well-being [21,22]. The outcome of resilience is therefore the result of cumulative protective factors effectively countering cumulative risk factors that an individual is exposed to [23,24].

Social support, which refers to one being part of a supportive social network of other people who care and are available to help through life challenges, is key to sustaining mental well-being and has been shown to assist with victim coping after experiencing a traumatic event [25–27]. Social support, or the lack of it, is important in enabling or hindering perpetrators’ ability to sustain abusive relationships through social isolation of victims. Levels of social support are lower among abused women compared to non-abused women [26,28]. Moreover, mental ill health symptoms – for example, depression and suicidality – are heightened among victims who are more socially isolated [26,29].

Among abused women, seeking support from a network of people who are supportive and sympathetic enhances women’s coping skills and mitigates the adverse mental health outcomes of IPV [26,28–30]. On the contrary, in many instances women victims of abuse receive negative reactions from social resources who may blame, or be unsupportive. This is not beneficial and may pose as a further deterrent to women seeking help [28]. The availability of positive social support is associated with women’s greater access and utilization of services. Abused women who lack positive social support tend to be less knowledgeable about available formal support systems, and are less likely to utilize formal services [28]. However, the relationship of positive social support and service utilization is confounded by socio-economic status, in particular the lack of financial resources or low accessibility of services that is common in low-income settings [28,31]. The utilization of formal services is also influenced by severity and frequency of abuse, availability of personal resources, perceived efficacy and reactions of formal support systems [28,31,32].

The persistence of negative societal attitudes that seek to maintain traditional gender roles and justifiy male dominance over women also impacts negatively on abused women’s willingness to disclose violence experiences and seek social support [31]. When severe abuse is met with stigma in the community, women will be less likely to disclose it to family and even less likely to disclose outside the family, or report it to police or other formal service providers [31].

The body of knowledge on resilience among abused African women is limited, yet it is important to understand why some abused women are resilient while others develop mental ill health. Fewer studies have explored the mediating effects of social support, use of services and self-disclosure of abuse in reducing the risk of mental ill health amongst abused women. In the present study, we took the absence of psychopathology to be an indicator of resilience. This paper describes the characteristics of abused women who did not report PTSD or depressive symptoms and distinguishes them from those who were abused and reported symptoms. We also present factors associated with the absence of depressive and PTSD symptoms among abused women.

## Methods

We used data collected through a household survey with a representative sample of women from Gauteng Province of South Africa. The survey design included a multi-stage random sampling approach to randomly select 40 enumeration areas (EA) at the first stage. At the second stage, 20 households were randomly selected in each EA and lastly only one adult woman residing in a selected household was invited to participate in the study. Researchers reached 96% of the selected households and 89% of these households had an eligible participant. The response rate among women was 79%. Interviews were conducted with 511 eligible women who gave written consent to participate in the study [33]. In this secondary analysis, only women who had experienced sexual or physical IPV in their lifetime were included (N = 189).

We obtained ethical approvals for the studies from the University of the Witwatersrand Faculty of Health Science Human Ethics Committee and the South African Medical Research Council Ethics Review Committee. All research was conducted conforming to safety guidelines for conducting research on domestic violence [34,35]. Researchers allocated anonymous study identification numbers to participants, conducted interviews in privacy and assured them of confidentiality including that no data would be linked back to them. Women also received local referrals for support when they requested it from the researchers.

### Measurement

#### Outcome variable

The main outcome of the study was resilience to PTSD and/or depression among abused women. Depression was measured using 20 items of the Centre for Epidemiologic Studies Depression Scale (CES-D) (Cronbach’s alpha = 0.942) [36]. We summed up the item scores to create a continuous CES-D score, then we used 16 + a cut-off to dichotomize the score. A score equal to or greater than 16 indicated a high probability of clinical depression. PTSD symptoms were measured using 30 items of the Harvard Trauma Questionnaire (Cronbach’s alpha = 0.975) [37]. Researchers asked participants whether each of the symptoms had bothered them in the past weeks and they could respond by ‘not at all’ (1), ‘a little’ (2), ‘quite often’ (3) and ‘extremely often’ (4). We summed scores for all items divided by 30 items to obtain a PTSD score and a 2.5 cut-off to indicate PTSD symptoms. We created a resilience variable by combining the depression and PTSD variables. Consistent with other studies, resilience was defined as having less than the threshold for depressive (CESD score < 16) and PTSD symptoms (PTSD score < 2.5) (24).

#### Exposure variables

The main exposure variables of interest were physical and/or sexual IPV in lifetime. Physical and/or sexual IPV experiences were measured using the World Health Organization (WHO) Multi-Country Study on Women’s Health and Domestic Violence: Core Questionnaire and WHO Instrument – Version 9 [38]. Physical IPV was defined as being slapped, having dangerous objects thrown at you, being pushed, kicked, hit, dragged, choked, beaten, burnt, or threatened by an intimate partner with a weapon. Sexual IPV was defined as being physically forced to have non-consensual sex or sex because of fear of what a male partner might do or being forced to do something sexual by a male partner that they found degrading or humiliating. Only women who had experienced acts of physical or sexual IPV were included in the study. Women’s experiences of IPV in the 12 months before the survey were generated using a recode of responses to the questions ‘Have any of these acts happened in the past 12 months?’ which followed the physical and sexual IPV item questions.

Child sexual abuse was measured by four items from the Childhood Trauma Questionnaire [39]. The items included ‘Before I turned 18 someone touched my buttocks or genitals or made me touch them when I did not want to’; ‘I had sex with a man who was more than 5 years older than me’; ‘I had sex with someone because I was threatened or frightened or forced and was forced to have sex against my will by a boyfriend.’ Possible responses for items of the Childhood Trauma Questionnaire were ‘never true’, ‘sometimes true’, ‘often true’ and ‘very often true’. We created dichotomous variables of never vs ever.

Lifetime experience of non-partner rape was measured by the question ‘How many times have you been forced or persuaded to have sex against your will by a man who wasn’t your husband or boyfriend?’ Possible responses were ‘never’, ‘once’ and ‘more than once’. We created a binary variable of never vs once or more times.

Other experiences of life traumatic events were measured using the Life Events checklist [40]. Traumatic events included imprisonment/detainment, civil unrest/war, serious injury requiring hospitalization, being close to death, witnessing a murder of family or friend, unnatural death of family or friend, witnessing the murder of stranger/s, torture, being robbed or carjacked at gun- or knifepoint and kidnapping. Responses to each trauma event were either ‘yes’ (1) or ‘no’ (0). We summed the responses to create a continuous trauma score and a binary outcome of no traumatic events (score = 0) vs one or more traumatic events exposures (score> 0).

Socio-demographic variables measured were age grouped into three categories: 18–29, 30–44 and 45+; education grouped into three categories: primary school and lower, secondary education and tertiary education, and employment in the year before the survey. Current relationship status was measured by the question ‘Do you currently have a husband or boyfriend?’ Alcohol consumption was measured using items from the Alcohol Use Disorders Identification Test (AUDIT) scale [41]. Researchers asked the participants whether they had drunk alcohol in the last year and whether they had consumed more than five drinks on one occasion.

Socio-economic status-related questions asked included ‘Would you say people in your home often, sometimes, seldom or never go without food?’ We created a binary variable of sometimes or often vs seldom or never. We also created a variable that combined employment status and income; i.e. no employment, employed and earning less than 2000R and earning more than 2000R.

Several social-support related questions were asked including ‘If you had an emergency and 200 South African Rands (equivalent of USD8) was needed immediately would you say it would be very easy, easy, quite difficult or very difficult to find the money?’ We created a binary category of easy or very easy vs quite or very difficult. This variable was used as an indicator for social connectedness and perceptions of social resources available in emergency.

Seeking family support was measured by variables that assessed self-disclosure of IPV experiences and seeking help from family when facing emotional difficulties. Questions included ‘Did you tell anyone in your family about abuse incidents?’ and ‘When feeling sad, disappointed or frustrated have you ever sought help from (1) older relatives (2) through family meetings?’ Possible responses were ‘yes’ or ‘no’. Women who sought help were also asked questions about how the family members responded. Possible negative responses were: they blamed me, they were indifferent and they told me to keep it quiet. Positive responses were: they supported me, they were hurt by it and they advised me to report to police. We created a categorical variable for not seeking support, seeking family support and receiving either positive or negative reactions. Informational support was measured by a proxy question: ‘How often have you talked to someone about domestic violence?’ Possible responses were: never, once or twice, sometimes and often. We created a binary variable of never talked about IPV vs. talked about IPV once or more.

Formal support services available to support abused women include medical and/or psychosocial services. We therefore assessed women’s utilization of services through questions about whether they had sought medical attention, had been to a shelter for abused women or received counselling for the abuse. Women were also asked the question ‘When feeling sad, disappointed or frustrated have you ever sought help from (1) professional counselling or (2) a doctor?’ We created a binary service utilization variable of using none of the services vs using one or more services.

Women’s perceptions of community attitudes towards gender relations and intimate partner violence were used as a proxy for cultural beliefs and perceived community support. Attitudes were measured by six questions with Likert responses of ‘strongly agree’ (1), ‘agree’ (2), ‘disagree’ (3) and ‘strongly disagree’ (4) (Cronbach’s 0.84). Examples of questions were ‘My community thinks that if a wife does something wrong, her husband has the right to punish her’; ‘My community thinks that if a man beats you, it shows he loves you’ and ‘My community thinks that if a man has paid lobola (bride price) for his wife, she must have sex whenever he wants it.’ The scores from the six items were summed up and scores ranged between 6 and 24 with lower scores indicating that women perceived their communities to be supportive of inequitable gender dynamics within intimate relationships and condoning or legitimizing the use of violence by male partners to control women. We created a binary variable of unsupportive vs supportive community perceptions using a cut-off of 15.

#### Data analysis

All analyses were done in Stata using ‘svy’ commands taking into account the multi-stage sampling design of the survey. We conducted bivariate analysis by cross-tabulation of variables by the resilience outcome variable to describe the prevalence of resilience by the different characteristics (Table 1). Secondly, we built a multivariable logistic regression model and included socio-demographic variables that had a p-value less than 0.2 in the bivariate analyses and all the explanatory variables that were associated with resilience in literature. (Table 2). Socio-demographic variables included in the regression model included employment and income, alcohol consumption, utilization of medical or psychosocial services and severity of IPV in the past 12 months. Social support variables were ease of finding money in emergency, family support and reactions, knowing someone who is abused in the community or family and talking about IPV. We adjusted the model for the traumatic exposures, namely history of child sexual abuse, non-partner rape and other traumatic events.

**Table 1.** Resilience disaggregated by socio-demographic, trauma exposures and social support.

	PTSD or depression symptoms		Resilient (below threshold for PTSD or depression)		Total		
	N	%	N	%	N	%	P value
**Age group**							
18–29	33	30.3	24	30.0	57	30.2	0.859
30–44	41	37.6	33	41.3	74	39.2	
45+	35	32.1	23	28.8	258	30.7	
**Education**							
Primary schooling and below	21	19.3	17	21.3	38	20.1	0.936
Secondary schooling	74	67.9	53	66.3	127	67.2	
Tertiary education	14	12.8	10	12.5	24	12.7	
**Relationship status**							
Not in relationship	25	22.9	18	22.5	43	22.8	0.015
In relationship but not cohabiting	43	39.5	16	20.0	59	31.2	
In relationship and cohabiting	41	37.6	46	57.5	87	46.0	
**Employment and income**							
Not employed	63	57.8	48	60.0	111	58.7	0.317
Employed earned < 2000R	17	15.6	17	21.3	34	18.0	
Earned more than 2000R	29	26.6	15	18.8	44	23.3	
**No alcohol consumption in past 12 months**	49	45.0	50	62.5	99	52.4	0.017
Alcohol consumed, no binge drinking	27	24.8	18	22.5	45	23.8	
Binge drinking	33	30.3	12	15	45	23.8	
**Difficult to find money in emergency**	94	86.2	57	71.3	151	79.9	0.031
Easy to find money in emergency	15	13.8	23	28.8	38	20.1	
**Family often or sometimes without food**	49	45.0	34	42.5	83	43.9	0.772
Family seldom or never without food	60	55.1	46	57.5	106	56.1	
**History of child sexual abuse**							
Never	63	57.8	54	67.5	117	61.9	0.184
Once or more	46	42.2	26	32.5	72	38.1	
**Non-partner rape**							
None	78	72.9	66	83.5	144	77.4	0.058
Once or more	29	27.1	13	16.5	42	22.6	
**Other life traumatic event**							
None	43	40.2	42	52.5	85	45.5	0.100
One or more	64	59.8	38	47.5	102	54.6	
**Perceptions of community attitudes**							
Unsupportive	65	59.6	37	46.3	102	54.0	0.091
Supportive	44	40.4	43	53.8	87	46.0	
**Family support**							
No	26	23.9	28	35.0	54	28.6	0.039
Yes	83	76.2	52	65.0	135	71.4	
**Medical or psychosocial services utilization**							
No	58	53.2	56	70.0	114	60.3	0.006
Yes	51	46.8	24	30.0	75	39.7	
**Physical, sexual or emotional IPV in past 12 months**							
None	53	48.6	57	71.3	110	58.2	0.014
Once or more	56	51.4	23	28.8	79	41.8	
**Severity/frequency of IPV**							
Mild	65	59.6	57	71.3	122	64.6	0.149
Severe	44	40.4	23	28.8	67	35.5	
**IPV gradient**							
No IPV in last 12 months	53	48.6	57	71.3	110	58.2	0.011
Mild IPV in past 12moths	28	25.7	15	18.8	43	22.8	
Severe (repeat sexual and physical) IPV in past 12 months	28	25.7	8	10.0	36	19.1	
**Know someone else abused by partner**							
No	28	25.7	32	40.0	60	31.8	0.047
Yes	81	74.3	48	60.0	129	68.3	
**Talk about IPV**							
No	52	48.2	37	46.3	89	47.3	0.779
Yes	56	51.9	43	53.8	99	52.7

**Table 2.** Factors associated with resilience among abused women.

	AOR	95% CI		P value
**Unemployed in past 12 months**	1.00			
Employed earned < 2000R	1.75	0.49	6.27	0.375
Earned more than 2000R	0.36	0.14	0.91	0.033
**No alcohol consumption in past 12 months**	1.00			
Alcohol consumed, no binge drinking	0.54	0.21	1.39	0.195
Binge drinking	0.39	0.17	0.91	0.03
**No IPV in past 12 months**	1.00			
Mild IPV	0.57	0.21	1.53	0.253
Severe IPV	0.29	0.12	0.74	0.011
**Other trauma exposure**	0.88	0.40	1.94	0.744
**History of child sexual violence**	1.32	0.61	2.82	0.468
**Experienced non-partner rape once or more in lifetime**	0.62	0.24	1.61	0.311
**Difficult to find money in emergency**	1.00			
Easy to find money in emergency	5.37	1.63	17.64	0.007
**Perceptions of supportive community**	2.35	1.03	5.35	0.043
**No family support sought**	1.00			
Family support and supportive reaction	0.62	0.30	1.27	0.181
Family support and negative reaction	0.22	0.05	0.98	0.047
**Used medical or psychosocial services**	0.48	0.28	0.80	0.006
**Knowing someone who is abused in community or family**	0.46	0.19	1.11	0.082
**Talked to someone else about IPV**	1.29	0.59	2.80	0.508

## Results

The sample comprised 189 women who experienced physical or sexual IPV in their lifetime. Forty-two percent of women in the sample experienced IPV in the year before the survey. Table 1 shows that most of the women were less than 44 years of age (69.4%), had attended secondary education (80%), had not worked in the past year (56.6%) and were in a current intimate relationship (77.3%). The majority of women found it difficult to obtain 200 South African Rands in an emergency (79.9%) and close to half (44%) did not have food in their households. Twenty-two percent of women had been raped by a non-partner, 38.1% had experienced child sexual abuse and 54.6% had experienced other traumatic events. Over half of women (51.3%) consumed alcohol and 24.7% binge drank in the past year.

The majority of women sought family support (71.4%) and more women received supportive reactions than negative reactions. Fewer women utilized medical or psychosocial services (40%) or reported to police (18.5%). About half of the women perceived their communities to be more supportive of equitable gender norms (46%). Almost two-thirds of the women knew another abused woman in their community or family and over half of the women had talked to someone about domestic violence (52.7%) (Table 1)

Forty-two percent of women scored below the threshold for PTSD or depressive symptoms at the time of the survey and so were categorized as resilient. Fifty-eight percent of the women scored above the thresholds: 56% of the women scored above the 16+ threshold for depression and 15.4% had above the 2.5 threshold on the PTSD scores. Forty-two percent of the women had depression symptoms only, 1.1% women had PTSD symptoms only and 14.3% had both depression and PTSD symptoms.

A significantly higher proportion of women who were in current intimate relationships and cohabiting compared to those who were not in relationships (57.5% vs 37.6%) were resilient. Furthermore, more women who perceived that their community was supportive were resilient compared to women who perceived their community to be unsupportive (53.8% vs 40.4%). A higher proportion of women who found it easy to find money in an emergency were resilient compared to those women who found it difficult (28.8% vs 13.8%). Women who experienced IPV in the 12 months before the survey (28.7% vs 51.4%), knew some other woman who was abused (60% vs 74.3%), who sought family support (65% vs 76.2%) or utilized services (30% vs 46.8%) were less likely to have scores less than the threshold for PTSD or depressive symptoms. There were no significant differences in the proportion of non-resilient vs resilient women by age group, level of education, employment status, availability of food in the home and experiences of child sexual abuse, non-partner rape or other traumatic event exposure. Almost similar proportions of resilient and non-resilient women had talked to someone about domestic violence (Table 1).

Table 2 shows that women who binge drank, had unsupportive reactions from family, used medical or psychosocial services or experienced repeat IPV episodes in the past year were less likely to be resilient. Women who worked and earned over R2000 a month were less likely to be resilient compared to unemployed women (Adjusted Odds Ratio [AOR] 0.36; 95% CI 0.14–0.91). Women who binge drank were less likely to be resilient compared to women who had not drunk alcohol in the past year (AOR 0.39; 95% CI 0.17–0.91). Women who received negative family reactions were less likely to be resilient compared to women who did not seek family support (AOR 0.22 95% CI 0.05–0.98). Increased resilience was associated with perceptions of a supportive community (AOR 2.35; 95% CI 1.03–5.35) and being able to find money in an emergency (AOR 5.37; 95% CI 1.63–17.64).

## Discussion

This study shows evidence that among women who experienced sexual or physical IPV in their life, about half of the women did not report symptoms of PTSD or depression and so were considered to be resilient. Severity of IPV was associated with resilience, and women who experienced more severe IPV in the past 12 months were less likely to be resilient. This is consistent with research that shows a strong dose-response relationship between severity of abuse and mental ill health [27,42–47]. Other research has shown that due to social isolation by perpetrators, women who experience severe IPV are less likely to disclose abuse and receive social support from their informal networks or from formal services [48].

After adjusting for IPV severity and violent exposures, the main factors that were associated with increased resilience were indicators of social support. Women’s social capital and connectedness influence their ability to mobilize resources from their networks and have been identified as associated with positive effects on health outcomes [49]. More of the women in this study said they would find it difficult to get money in an emergency. This may be indicative of their poor social connectedness, weaker social ties or limited practical support available from their support networks. In contrast, being able to find money in an emergency was a proxy indicator that women are sufficiently socially connected or have practical social support. We found that the women who found it easier to find money in an emergency were more likely to be resilient and this could relate to both resource access and stronger social ties. Stronger social ties are associated with greater disclosure to informal networks coupled with the greater practical support that may result in greater access to formal support [28,50].

The social context in which abused women live impacts on their connectedness to others and their access to formal services and ultimately on their ability to cope in or leave abusive relationships (50). Inherently, the social contexts in which women live can vary substantially. Some communities have greater social cohesion than others. Prevailing community attitudes play an important role in responses to violence against women [51]. In communities with less social cohesion and where inequitable gender and subjective norms are prevailing, people are more likely to justify the abuse of women, and victims will be less likely to disclose abuse or seek help [52]. Conversely, abused women living in supportive communities are more likely to disclose abuse compared to women living in unsupportive communities where women are blamed for the abuse [29]. However, the beneficial health outcomes of social capital occur at the individual level and so may vary among individuals even within the same community [53]. When individuals perceive their communities to be supportive towards victims of abuse, this has benefits for the mental health of individuals, making them more likely to be psychologically resilient as shown in this study [24,54].

Similar to many studies, most women in the study sought family support for emotional difficulties and disclosed abuse to family members [28]. Disclosing abuse and seeking help from formal support services has been shown to avert the long-term mental health impact of abuse. Consistent with other literature, seeking family support in itself was not associated with a positive impact on mental health but rather the positive effect was dependent on positive reactions of the people the women disclosed to [28,29]. In this study, women who received a negative family reaction were less likely to be resilient. Research has shown that women who receive negative reactions when disclosing violence feel revictimized [28,55].

The utilization of formal support services increases with available resources (for example, having transportation access), the severity of the abuse and its association with psychiatric or mental illness, community attitudes towards victims, and previous trauma history [24,29,44,56–60]. In our study, we found that generally a smaller proportion of women consulted mental health practitioners, indicating the presence of barriers in women’s access to services. Contrary to the expected positive mental benefits of service utilization, we found that the fewer women who utilized services were less likely to be resilient. This finding should be further investigated as it may point to the limited efficacy of the services provided to victims. It is a possibility that interventions could be symptom focused and not address other needs of women such as the continued exposure to violence and thus have short-lived benefits [61,62].

Among abused women, heavy drinkers have been found to report more severe symptoms, suggesting that the relationship of trauma, PTSD and alcohol use could be motivated by reasons such as ‘drinking to cope’ [46]. However, in this study, women’s binge drinking as a coping strategy did not confer increased resilience.

Women who were employed and earned more were less likely to be resilient, a deviation from other studies showing that higher socio-economic status is associated with being more resilient [42,63]. This observation may be explained by the differential access to informal social support and coping resources available to women that may supersede the ownership of material resources in conferring resilience.

This study is not without limitations. The restriction of the survey data to only abused women resulted in a smaller sample size for analysis. This may have limited study power or affected the associations and effect sizes against variables. Smaller studies sometimes show different, often larger effects than large ones. Moreover, the analysis is based on individual factors and did not measure other factors such as biological, environmental and societal factors that play a role in the development of psychological resilience. As such, the conclusions made in this study only pertain to the observed associations. Other social support measures could also be used in addition to longitudinal research which may be more appropriate in investigating a phenomenon such as resilience that is not static and can change in response to different life stressors over time. This study was limited in that it was cross-sectional and focused on resilience at one time point. As a result, we cannot be sure of the direction of some relationships. For example, the association between alcohol use and mental ill health may be bidirectional. Pathways to resilience differ at an individual level and may not always lead to the absence of psychopathology [23,24]. However for an individual exposed to violent exposures it was appropriate to define resilience in terms of the absence of psychiatric diagnoses [18,24]. In this study, we took the approach that resilience is the absence of psychopathology but future research could explore other definitions and indicators for resilience including using standardized resilience scales among abused women to expand the body of knowledge for the field in African settings.

## Conclusion and policy implication

Notwithstanding, our findings suggest that social support indicators including social connectedness, stronger network ties and perceived supportive communities are key factors in fostering resilience among abused women. Negative family reactions negatively impacted women’s mental health, and accessing formal services was not positively associated with resilience. In the study settings where mental health services are limited and often not accessed as shown in this study, the value of family or community-based support cannot be overemphasized. In addition to interventions to increase women’s utilization of available services, there is a need to promote stronger social support and social networks in families and communities. Support groups can be platforms through which women network with other women who are abused and opportunities to obtain instrumental support that could help them cope better as well as foster self-efficacy and esteem [55]. Capacity-building for community-based organizations to provide effective social support within the reach of women is also critical. Further research on social support and other protective factors is necessary using bigger samples and longitudinal design to inform context-specific and targeted interventions that work.

## Paper context

Extant literature has shown the potential benefits of social support in fostering women victims’ psychological resilience. The current study extends existing literature using data from African women who live under circumstances of economic and social disadvantage. The findings show that social support is integral to reducing the risk of mental ill health among African women. Education and capacity-building for family and community organizations to provide effective social support within the reach of women should be prioritized.
